# Supplementary material for: Mosquito Vector Competence for Japanese Encephalitis Virus
Source: Viruses. 2021 Jun 16;13(6):1154. doi: 10.3390/v13061154 (PMC8234777; doi:10.3390/v13061154)
Supplement: Supplementary file 1 [file viruses-13-01154-s001.zip › viruses-1239479-supplementary.pdf]

Table S1. Mosquito vector competence studies for JEV.

Mosquito Species	Ref.	Mosquito Origin *	Virus Strain &	Virus Concentration	Mosquito Infection Procedure	Mosquito Infection Rate	Mosquito Dissemination Rate	Mosquito Transmission Rate #
Aedes aegypti	[181]	Taiwan (laboratory colony, Tainan)	n/d (isolated from *Cx. annulus*, Taiwan 1972)	$10^{4.2}$ MID50 per mosquito	Parenteral infection (injection)	JEV detected at 7 dpi	n/d	n/d
	[162]	n/d (laboratory colony, CDC)	SA14 (genotype III; Isolated from *Cx. pipiens*, China, 1954)	6.0 $\log_{10}$ pfu/mL (5.5 $\log_{10}$ pfu per mosquito) for intrathoracic injection	Intrathoracic injection and feeding blood meal with washed calf red blood cells with cotton pledgets for 15–30 min	*Intrathoracic injection:* at 9 dpi 6.3 $\log_{10}$ per mosquito; *Orally infected:* at 15 dpi 5.4 $\log_{10}$ pfu per mosquito	n/d	n/d
	[38]	Australia (laboratory colony, Townsville, Queensland)	TS3306 (genotype II; *Och. vigilax,* Australia, 1998)	mean $10^{4.5}$ TCID50/mL	feeding for 2–4 h at 37 °C on virus diluted in heparinized rabbit blood and 1% sucrose by glass membrane feeder (pig intestine as membrane)	27% (16/60) at 14–15 dpi	17% (10/60) [legs], 25% (15/60) [heads] at 14–15 dpi	n/d
	[168]	n/d (laboratory colony)	N28 (genotype III; pig isolate China, 2015)	10^3 MID50	Intrathoracic injection or blood meal feeding of mice blood with Hemotek system	JEV detectable at 10 dpi in whole body and midgut	n/d	JEV detectable at 10 dpi in salivary glands
Aedes albolineatus	[181]	Taiwan (laboratory colony, Nantou)	n/d (isolated from *Cx. annulus*, Taiwan 1972)	$10^{4.2}$ MID50 per mosquito	Parenteral infection (injection)	JEV detected at 7 dpi	n/d	n/d
Aedes albopictus	[161]	Taiwan (laboratory colony, Taipei, 1960)	T-143 (*Cx. tritaeniorhynchus*, Taipei, Taiwan, 1960)	viremia in pig: 10^1–$10^{2.5}$ LD50	feeding on viremic pig	0% (0/114)	n/d	n/d
	[181]	Taiwan (laboratory colony, Taipei)	n/d (isolated from *Cx. annulus*, Taiwan 1972)	$10^{4.2}$ MID50 per mosquito	Parenteral infection (injection)	JEV detected at 7 dpi	n/d	n/d

	[31]	Taiwan (laboratory colonies from Sanhsia, Yungho, and Liyang)	MQ1–2 (*Ae. albopictus*, Taipei, Taiwan, 1985)	10-fold serial dilutions of 7.5 log10 WMICLD50/ 0.03 mL	intrathoracic injection, or ingestion (hanging drop feeding of defibrinated rabbit blood and 5% sucrose)	Virus conc. in mosquitoes from Sanhsia: 2.03 log10 MID50; Yungho: 4.32 log10 MID50; Liyang: 4.98 log10 MID50	n/d	27–39% with Sanhsia mosquitoes only (transmission to weanling mice)
	[162]	n/d (laboratory colony, CDC)	SA14 (genotype III; Isolated from *Cx. pipiens*, China, 1954)	6.0 log_{10} pfu/mL (5.5 log_{10} pfu per mosquito) for intrathoracic injection	Intrathoracic injection and feeding blood meal with washed calf red blood cells with cotton pledgets for 15–30 min	*Intrathoracic injection:* at 18 dpi 6.3 log_{10} per mosquito;	n/d	n/d
	[32]	Taiwan (Liu-Chiu)	CH1392 (genotype III; *Cx. tritaeniorhynchus*, Taiwan, 1990)	1.25 × 10^7 PFU/mL	fed on drop of rabbit blood (3 mL rabbit blood, 0.3 g sucrose, 1 mL virus suspension) for 1 h	n/d	n/d	47% at 14 dpi [virus detection in salivary glands]
	[33]	Australia (field caught, F7, Masig Island, 2005)	TS00 (genotype I: pig, Australia, 2000)	3.5 log10 TCID50/mL per mosquito	Feeding virus diluted in defibrinated sheep blood and 1% sucrose over cotton pledget for 2–4 h	20% (4/25) at 14 dpi	16% (4/25) at 14 dpi	16% (4/25) at 14 dpi
	[34]	France (Nice, 2011)	recombinant virus from strain RP-9 (genotype III; wild type strain NT109 isolated from *Cx. tritaeniorhynchus*, Taiwan, 1985); And from strain XZ0934 (genotype V; isolated from *Cx. tritaeniorhynchus*, China, 2009)	8 × 10^6 FFU/mL	blood meal of washed rabbit erythrocytes with 5µM ATP, fed for 2 h via blood-soaked cotton pledges in the dark at 28˚C	57–90% at 7 dpi 90–100% at 11–13 dpi	60–90% at 7 dpi 90–100% at 11–13 dpi (measured as infected heads)	20–63% at 11–13 dpi (mean conc. in saliva RP-9: 45 FFU/saliva; XZ093: 38 FFU/saliva)
Aedes alcasidi	[181]	Taiwan (laboratory colony, Taitung)	n/d (isolated from *Cx. annulus*, Taiwan 1972)	$10^{4.2}$ MID50 per mosquito	Parenteral infection (injection)	JEV detected at 7 dpi	n/d	n/d
Aedes desmotes	[181]	Taiwan (laboratory colony, Chiai)	n/d (isolated from *Cx. annulus*, Taiwan 1972)	$10^{4.2}$ MID50 per mosquito	Parenteral infection (injection)	JEV detected at 7 dpi	n/d	n/d

Aedes detritus	[111]	United Kingdom (England)	Muar (genotype V; human, Malaysia,1952)	6 × log10 pfu/mL	Feeding defibrinated horse blood supplemented with 0.02 µM ATP via Hemotek membrane feeding system (Parafilm as membrane)	23 °C:<br>32% (8/25) at 7 dpi,<br>78% (25/32) at 14 dpi,<br>100% (6/6) at 21 dpi<br>28 °C:<br>60% (9/15) at 7 dpi,<br>50% (3/6) at 14 dpi,<br>100% (3/3) at 21 dpi	23 °C:<br>20% (5/25) at 7 dpi,<br>72% (23/32) at 14 dpi,<br>100% (6/6) at 21 dpi<br>28 °C:<br>60% (9/15) at 7 dpi,<br>33% (2/6) at 14 dpi,<br>100% (3/3) at 21 dpi	23 °C:<br>12% (3/25) at 7 dpi,<br>3% (1/32) at 14 dpi,<br>67% (4/6) at 21 dpi<br><br>28 °C:<br>27% (4/15) at 7 dpi,<br>17% (1/6) at 14 dpi,<br>33% (1/3) at 21 dpi
Aedes dorsalis	[36]	USA	Nakayama (genotype III; human, Japan, 1935)	n/d	feeding on mouse brain virus suspension and defibrinated rabbit blood presented over cotton moistened with mixture of 10%	no infection detected (no virus recovery from blood-fed mosquitoes)	n/d	17% (1/6) at 16 dpi [transmission to 21–28 day old Swiss mice (Webster strain)]
Aedes japonicus	[23]	Japan (Sapporo)	JANAr-5681 (*Cx. tritaeniorhynchus*, Japan, 1981)	$10^{6.2}$ pfu/mL (*blood-soaked cotton*)<br>$10^{3.7}$ pfu/mL (*viremic chick*)	virus-blood mixture fed over soaked cotton or viremic chick	feeding on *blood-soaked cotton*:<br>90% at 14 dpi<br>*viremic chick*:<br>45% at 14 dpi	n/d	*blood-soaked cotton*:<br>75% at 14 dpi<br>*viremic chick*:<br>33% at 14 dpi<br>[transmission to mice]
	[175]	Germany (Stuttgart) [*Aedes japonicus japonicus*]	n/d	n/d	60% human blood, 30% fructose solution, 10% fetal calf serum, fed for 3 h on a soaked cotton stick	100% at 14 dpi	n/d	n/d

	[157]	Japan (laboratory colony, Chiba prefecture, 2004) [*Aedes japonicus japonicus*]	17CxIT-I4-D31 (genotype I, *Cx. tritaeniorhynchus*, Japan, 2017); JaGAr01 (genotype III, *Cx. tritaeniorhynchus*, Japan, 1959); Muar (genotype V, human, Malysia, 1952)	17CxIT-I4-D31: 8.9 log10 ffu/mL; JaGAr01: 8.6 log10 ffu/mL; Muar: 7.1 log10 ffu/mL	Feeding defibrinated rabbit blood 1:1 mixed with virus, supplemented with 3 mM ATP with Hemotek feeder (pig intestine as membrane)	17CxIT-I4-D31: 12% (3/25) at 7 dpi, 0% (0/23) at 14 dpi; JaGAr01: 22% (7/32) at 7 dpi, 16% (5/32) at 14 dpi; Muar: 0% (0/360 at 7 dpi, 3% (1/29) at 14 dpi	17CxIT-I4-D31: 12% (3/25) at 7 dpi, 0% (0/23) at 14 dpi; JaGAr01: 22% (7/32) at 7 dpi, 16% (5/32) at 14 dpi; Muar: 0% (0/360 at 7 dpi, 3% (1/29) at 14 dpi	17CxIT-I4-D31: 67% (2/3) at 7 dpi; JaGAr01: 71% (5/7) at 7 dpi, 100% (5/5) at 14 dpi; Muar: 100% (1/1) at 14 dpi
Aedes kochi	[38]	Australia (Queensland, Bamaga and Cairns)	TS3306 (genotype II; isolated from *Och. vigilax,* Australia, 1998)	mean $10^{4.5}$ TCID50/mL	feeding for 2–4 h at 37°C on virus diluted in heparinized rabbit blood and 1% sucrose by glass membrane feeder (pig intestine as membrane)	Bamaga: 21% (6/28) at 14–15 dpi Cairns: 11% (1/9) at 14–15 dpi	n/d	Bamaga: 0% (0/8) at 14–15 dpi Cairns: 13% (1/8) at 14–15 dpi [transmission to Quackenbush suckling mice]
Aedes malikuli	[181]	Taiwan (laboratory colony, Chiai)	n/d (isolated from *Cx. annulus*, Taiwan 1972)	$10^{4.2}$ MID50 per mosquito	Parenteral infection (injection)	JEV detected at 7 dpi	n/d	n/d
Aedes nigromaculis	[36]	USA	Nakayama (genotype III; human, Japan, 1935)	n/d	feeding mixture of 10% mouse brain virus suspension and defibrinated rabbit blood over cotton	infection proven due to virus recovery from blood-fed mosquitoes at 16 dpi	n/d	80% (4/5) at 8–14 dpi [transmission to 21–28 day old Swiss mice (Webster strain)]
Aedes notoscriptus	[38]	Australia (laboratory colony, Closeburn, Queensland; and field-caught, Cairns, Queensland)	TS3306 (genotype II; isolated from *Och. vigilax,* Australia, 1998)	mean $10^{4.5}$ TCID50/mL	feeding for 2–4 h at 37 °C on virus diluted in heparinized rabbit blood and 1% sucrose by glass membrane feeder (pig intestine as membrane)	colony: 27% (13/48) at 13–14 dpi field-caught: 20% (1/5) at 14–15 dpi	colony: 8% (4/48) at 13–14 dpi field-caught: 20% (1/5) at 14–15 dpi	colony: 27% (3/11) at 13–14 dpi [transmission to Quackenbush suckling mice]
	[166]	New Zealand (field-caught, Auckland, 2009)	Nakayama (genotype III; human, Japan, 1935)	8.3 log10 pfu/mL	Feeding of virus mixed with goose, rabbit, or bovine blood, 2.5% sucrose, 0.02 µM ATP over soaked pledget	0% (0/39) at 14 dpi	n/d	n/d

Aedes purpureus	[38]	Australia (field-caught, Bamaga, Queensland)	TS3306 (genotype II; isolated from *Och. vigilax,* Australia, 1998)	mean $10^{4.5}$ TCID50/mL	feeding for 2–4 h at 37 °C on virus diluted in heparinized rabbit blood and 1% sucrose by glass membrane feeder (pig intestine as membrane)	100% (2/2) at 14–15 dpi	100% (2/2) at 14–15 dpi [virus detected in head + salivary glands] but no virus detected in legs	n/d
Aedes togoi	[181]	Taiwan (laboratory colony, Taipei)	n/d (isolated from *Cx. annulus*, Taiwan 1972)	$10^{4.2}$ MID50 per mosquito	Parenteral infection (injection)	JEV detected at 7 dpi	n/d	n/d
Aedes varipalpus	[36]	USA	Nakayama (genotype III; human, Japan, 1935)	n/d	feeding on mixture of 10% mouse brain virus suspension and defibrinated rabbit blood via cotton	infection proven due to virus recovery from blood-fed mosquitoes at 14 dpi	n/d	no transmission to 21–28 day old Swiss mice (Webster strain)
Aedes vexans	[35,35]	Guam	Okinawa strain (human, Japan, 1945)	n/d	n/d	n/d	n/d	successful transmission to infant mice
	[36]	USA	Nakayama (genotype III; human, Japan, 1935)	n/d	feeding on soaked cotton moistened with mixture of 10% mouse brain virus suspension and defibrinated rabbit blood	no infection detected [no virus recovery from blood-fed mosquitoes]	n/d	no transmission to 21–28 day old Swiss mice (Webster strain)
Aedes vexans nipponii	[23]	Japan (Sapporo)	JANAr-5681 (*Cx. tritaeniorhynchus*, Japan, 1981)	$10^{4.7}$ PFU/mL	viremic chick (inoculated with virus at the age of 1 d and maintained for 48-72 h)	25% at 14 dpi	n/d	n/d
Aedes vigilax	[38]	Australia (laboratory colony, Redlands Shire, Qeensland; and field-caught, Cairns, Queensland)	TS3306 (genotype II; *Och. vigilax,* Australia, 1998)	mean $10^{4.5}$ TCID50/mL	feeding for 2–4 h at 37 °C on virus diluted in heparinized rabbit blood and 1% sucrose by glass membrane feeder (pig intestine as membrane)	colony: 19% (12/62) at 9 dpi 39% (5/13) at 13 dpi field-caught: 57% (43/75) at 14–15 dpi	colony: 18% (11/62) at 9 dpi 39% (5/13) at 13 dpi	colony: 0% (0/4) at 13 dpi field-caught: 17% (3/18) at 14–15 dpi
	[182]	Australia (laboratory colony)	Nakayama (genotype III; human, Japan, 1935)	6.7 $\log_{10}$ pfu/mL (intrathoracic injection: 0.15 µL; blood meal: ≈1.7 µL)	Intrathoracic injection or blood meal feeding with sheep erythrocytes through commercial sausage skin membrane at 37℃	*intrathoracic injection*: 88% (53/60) (6.2 $\log_{10}$ pfu/mosquito); *blood meal*: 12% (7/60)	n/d	n/d
Anopheles hyrcanus.	[176]	India (laboratory colony, Pune)	n/d	n/d	n/d	virus detected up to 11 dpi	n/d	no transmission to chicks

Anopheles freeborni	[36]	USA [*An. maculipennis freeborni*]	Nakayama (genotype III; human, Japan, 1935)	n/d	feeding mixture of 10% mouse brain virus suspension and defibrinated rabbit blood on soaked cotton	infection proven due to virus recovery from blood-fed mosquitoes at 0–8 dpi	n/d	no transmission to 21–28 day old Swiss mice (Webster strain)
Anopheles sinensis	[181]	Taiwan (Taipei)	n/d (isolated from *Cx. annulus*, Taiwan 1972)	$10^{4.2}$ MID50 per mosquito	Parenteral infection (injection)	JEV detected at 7 dpi	n/d	n/d
Anopheles stephensi	[181]	n/d	n/d (isolated from *Cx. annulus*, Taiwan 1972)	$10^{4.2}$ MID50 per mosquito	Parenteral infection (injection)	JEV detected at 7 dpi	n/d	n/d
Anopheles tessellatus	[110]	India (laboratory colony, 1968)	733913 (genotype I; human, India)	4.0 dex (viremia in chicks)	feeding on chicks (infected with 5.0 dex) over night	virus detected up to 11 days after infection	n/d	31% (4/13) at 6–11 dpi [transmission to chicks]
Armigeres subalbatus	[181]	Taiwan (laboratory colony, Taitung)	n/d (isolated from *Cx. annulus*, Taiwan 1972)	$10^{4.2}$ MID50 per mosquito	Parenteral infection (injection)	JEV detected at 7 dpi	n/d	n/d
	[32]	Taiwan (Liu-Chiu, 1997)	CH1392 (genotype III; *Cx. tritaeniorhynchus*, Taiwan, 1990) T1P1 (genotype III; *Arm. subalbatus*, Taiwan, 1997)	1.25 10^7 pfu/mL	feeding for 1 h on drop of rabbit blood (3 mL rabbit blood, 0.3 g sucrose, 1 mL virus suspension)	n/d	n/d	CH1392: 88% at 14 dpi for JEV T1P1: 40% at 15 dpi and 79% at 20 dpi for [virus detection in salivary glands]
	[42]	Taiwan (laboratory colony, Liu-Chiu, 1997)	T1P1 (genotype III, Ar. subalbatus, Taiwan, 1997)	4.5 × 10^8 pfu/mL (0.17 µL per mosquito)	Intrathoracic injection of *Wolbachia*-infected and *Wolbachia*-free mosquitoes	n/d	n/d	*Wolbachia-infected*: 77% (23/30); *Wolbachia-infected*: 73% (22/30) at 7 dpi [measured by IFA in salivary glands]
Culex annulirostris	[35,35]	Guam [*Cx. jepsoni*]	Okinawa strain (human, Japan, 1945)	n/d	n/d	n/d	n/d	successful transmission to infant mice

	[38]	Australia (laboratory colony, Brisbane, Queensland; and field-caught from Bamaga and Cairns, Queensland)	TS3306 (genotype II; *Och. vigilax,* Australia, 1998)	mean $10^{4.5}$ TCID50/mL	feeding for 2–4 h at 37 °C on virus diluted in heparinized rabbit blood and 1% sucrose by glass membrane feeder (pig intestine as membrane)	laboratory colony:<br>78% (14/18) at 5 dpi<br>89% (16/18) at 7 dpi<br>94% (17/18) at 10 dpi<br>100% (36/36) at 14 dpi<br>Bamaga:<br>100% (2/2) at 14–15 dpi<br>Cairns:<br>93% (51/55) at 14–15 dpi	laboratory colony:<br>6% (1/18) at 5 dpi<br>33% (6/18) at 7 dpi<br>78%(14/18) at 10 dpi<br>64% (23/36) at 14 dpi	laboratory colony:<br>24% (4/17) dpi<br>57% (8/14) at 10 dpi<br>81% (13/16) at 14 dpi<br>Bamaga:<br>0% (0/2) at 14–15 dpi<br>Cairns:<br>61% (14/23) at 14–15 dpi<br>[transmission to Quackenbush suckling mice]
	[182]	Australia (laboratory colony)	Nakayama (genotype III; human, Japan, 1935)	6.7 log_{10} pfu/mL (intrathoracic injection: 0.15 µL; blood meal: ≈1.3 µL)	Intrathoracic injection or blood meal feeding with sheep erythrocytes through commercial sausage skin membrane at 37 ℃	*intrathoracic injection*: 6.0 log_{10} pfu/mosquito<br>*blood meal*: 7% (4/60) (5.0 log_{10} pfu/mosquito)	n/d	n/d
	[44]	Australia (laboratory colony, Brisbane, 1998)	TS3306 (genotype II; *Och. vigilax*, Australia, 1998)	n/d	feeding on hanging drop of heparinized rabbit blood and 1% sucrose	n/d	n/d	60% (6/10) at 13 dpi [transmission to flying fox *Pteropus alecto*]
Culex annulus	[181]	Taiwan (laboratory colony, Hsinchu)	n/d (isolated from *Cx. annulus*, Taiwan 1972)	$10^{4.2}$ MID50 per mosquito	Parenteral infection (injection)	JEV detected at 7 dpi	n/d	n/d
Culex bitaeniorhynchus	[49]	India (laboratory colony from Virus Research Centre)	733913 (genotype I; human, India)	n/d (ducklings infected with 4.9–5.2 dex)	feeding in viremic ducklings (viremia 1–4.5 dex)	feeding on chicken with *viremia of 1dex*: 9% at 9–12 dpi;<br>viremia of 4.2 dex: up to 100% at 9–12 dpi	n/d	transmission to duckling (1/1)
	[50]	India (Pune)	733913 (genotype I; human, India)	n/d (chicks infected with 2.4–4.5 dex)	feeding in viremic chicks (viremia 3.1–4.7 dex)	47–53% at 10 dpi<br>62% at 12 dpi	n/d	64–75% at 10 dpi<br>89% at 12 dpi<br>total: 18/24<br>[transmission to chicks]
	[51,164]	India (laboratory colony)	733913 (genotype I; human, India)	n/d (chicks infected with 4.0 dex)	feeding on chicks (infected with 4.0 dex)	n/d	n/d	successful transmission to chicks
Culex fuscocephala	[60]	Thailand (Chiengmai valley)	BKM-984–70 (*Cx. fuscocephala*, Thailand, 1970)	average 8 pfu per mosquito	feeding on viremic white Leghorn chicks (infected with 1700 pfu 55 h before mosquito feeding)	95–100% at 10–27 dpi	n/d	10–20% at 10–27 dpi [transmission to chicks]

	[61]	Taiwan (JEVRU laboratory colony)	TaiAn 171 (pig, Taiwan, 1971)	$10^{-0.89}$–$10^{-1.91}$ mouse LD50 in pig serum on day of mosquito feeding	feeding on viremic pigs (infected with $10^{5.5}$ mouse LD50)	n/d	n/d	0% at 12 dpi 22% at 17 dpi 68% at 21 dpi [transmission to white leghorn chicks]
Culex gelidus	[19]	Malaysia strain (subcolony of WRAIR)	FM380 (*Cx tritaeniorhynchus*, Japan, 1950)	1,3 x 10^5 LD50/mL; 3,3 x 10^5 LD50/mL	feeding on viremic white Leghorn chicks (1–3 days old, infected with 100–10,000 mouse LD50 1–3 days before mosquito feeding)	43.7% (116/265) at 6–21 dpi	n/d	72–85% (total: 31/40) at 28 dpi [transmission to White leghorn chicks]
	[151]	Malaysia (laboratory colony)	FM380 (*Cx tritaeniorhynchus*, Japan, 1950)	n/d [viremia in chicks around 10^4 LD50 in suckling mice after intracranial inoculation)	3–7 days after emergence feeding on viremic chicks (around 10^4 LD50) for transmission to horses; feeding on infected horse for further transmission to chicks	n/d	n/d	0% (0/42) transmission to chicks when mosquitoes fed on infected horse
	[38]	Australia (field caught, Cairns, Queensland)	TS3306 (genotype II; *Och. vigilax,* Australia, 1998)	mean $10^{4.5}$ TCID50/mL	feeding for 2–4 h at 37 °C on virus diluted in heparinized rabbit blood and 1% sucrose by glass membrane feeder (pig intestine as membrane)	100% (4/4) at 14–15 dpi	n/d	100% (1/1) at 14–15 dpi [transmission to Quackenbush suckling mice]
	[182]	Australia (laboratory colony)	Nakayama (genotype III; human, Japan, 1935)	6.7 $\log_{10}$ pfu/mL (intrathoracic injection: 0.15 µL; blood meal: ≈1.9 µL)	Intrathoracic injection or blood meal feeding with sheep erythrocytes over soaked cotton pledgets	*intrathoracic injection*: 92% (55/60); *blood meal*: 18% (11/60) (5.5 $\log_{10}$ pfu/mosquito)	n/d	n/d
	[82]	India (laboratory colony, Pune)	P20778 (genotype III, human, India, 1958)	n/d	intrathoracic injection	JEV detectable from 4 dpi to 14 dpi	n/d	JEv detectable at 10 dpi and 14 dpi
Culex pipiens	[90]	n/d	Nakayama (genotype III; human, Japan, 1935)	n/d	feeding on mouse-brain-virus solution in rabbit blood over cotton pads	n/d	n/d	50% (2/4) at 9 dpi
	[183]	Japan (laboratory colony)	JaGAr 01 (genotype III; *Cx. tritaeniorhynchus*, Japan 1959)	n/d	intrathoracic injection	n/d	n/d	JEV detection in salivary glands at 19 dpi
	[166]	USA (laboratory colony, Pennsylvania, 2002)	Nakayama (genotype III; human, Japan, 1935)	8.1 log10 pfu/mL	Feeding of virus mixed with goose, rabbit, or bovine blood, 2.5% sucrose, 0.02 µM ATP over soaked pledget	10% (5/50) at 14 dpi	40% (2/5) at 14 dpi	0% (0/5) at 14 dpi

	[94]	China (laboratory colony)	SH7 (genotype I; *Cx. tritaeniorhynchus*, China, 2016), SH15 (genotype III; *An. sinensis*, China, 2016)	8.3–8.65 log TCID50/mL in blood meals (4.78–4.9 log TCID50/mL in engorged mosquitoes)	Hemotek membrane feeder or cotton pledge with defibrinated mice blood	for SH7: 43% (12/28) at 7 dpi, 38% (17/45) at 14 dpi;<br><br>for SH15: 58% (19/33) at 7 dpi, 44% (23/52) at 14 dpi	for SH7: 27% (3/11) at 7 dpi, 24% (4/17) at 14 dpi;<br><br>for SH15: 32% (6/19) at 7 dpi, 35% (8/23) at 14 dpi	for SH7: 17% (2/12) at 7 dpi, 29% (5/17) at 14 dpi;<br><br>for SH15: 0% (0/13) at 7 dpi, 36% (8/22) at 14 dpi
	[95]	UK (field caught, F1, Cheshire)	CNS138–11 (genotype II, human, Malaysia, 1999)	1×10^6 pfu/mL	Feeding of heparinised human blood (heated to 39℃) with Hemotek feeding system for up to 3 h	18 ℃: 100% (18/18) at 21 dpi	n/d	18 ℃: 72% (13/18) at 21 dpi
	[97]	China (laboratory colony)	SH7 (genotype I; *Cx. tritaeniorhynchus*, China, 2016)	100 pfu per mosquito	Intrathoracic injection	100% (5/5) at 10 dpi	100% (5/5) at 10 dpi [JEV detection in legs and heads]	100% (5/5) at 10 dpi [JEV detection in salivary glands]; 30% (6/20) [transmission to ducklings]
***Culex pipiens molestus*)**	[36]	USA (laboratory colonies from Oakland, California) [*Culex molestus*]	Nakayama (genotype III; human, Japan, 1935)	n/d	feeding on soaked cotton moistened with mixture of 10% mouse brain virus suspension and defibrinated rabbit blood	infection proven due to virus recovery from blood-fed mosquitoes at 3 and 16 dpi	n/d	27% (3/11) at 7–20 dpi transmission to 21–28 day old Swiss mice (Webster strain)
	[92]	Taiwan (Taipei, 1996; F40–43 generation)	Sanhsia MQ1–2 (*Ae. albopictus*, Taiwan, 1995)	$10^{4.5}$ PFU/mL (ingested $10^{5.54}$ PFU/mL per mosquito); 10^3 to 10^8 PFU/mL	feeding on hanging blood (defibrinated rabbit blood with 10% of sucrose) for 3 h	n/d	n/d	80–100% (total: 10/11) [transmission to mice]
	[93]	Uzbekistan (Tashkent, 2004; F1 generation)	ROK-2.0028 (*Cx. tritaeniorhynchus*, South Korea, 2000)	$10^{4.5}$–$10^{5.4}$ PFU/mL	feeding on leghorn chicks (2–4 days old, inoculated with 10^4 PFU 2–3 days before mosquito feeding)	56% at 16–17 dpi<br><br>47% at 19 dpi<br><br>51% at 25–27 dpi	26% at 16–17 dpi<br><br>25% at 19 dpi<br><br>26% at 25–27 dpi	8% at 19 dpi<br><br>8% at 25–27 dpi<br><br>[transmission to leghorn chicks]

Culex pipiens pallens	[169]	Japan (HRp laboratory colony)	JaGAr 01 (genotype III; *Cx. Tritaeniorhynchus,* Japan 1959); JaGAr 19461 (*Cx. tritaeniorhynchu*s, Japan 1961)	viremic chicken with 10^3 LD50/0.02 mL; or artificial blood meal with $10^{1.7}$–$10^{7.4}$ LD50/mL	feeding on viremic chicken or artificial blood meal with rabbit blood	JaGAr 01 at 10–14 dpi feeding on *viremic chicken*: 0% (0/23); *artificial blood meal* with $10^{1.7}$–$10^{2.2}$ LD50/0.02 mL: 0% (0/14) ; $10^{3.9}$–10^4 LD50/0.02 mL: 8% (1/12) JaGAr 19461 at 10–14 dpi after *artificial blood meal* with 10^4 LD50/0.02 mL: 15% (5/34); 10^5 LD50/0.02 mL: 68% (19/28) $10^{6.7}$–$10^{7.4}$ LD50/0.02 mL: 100% (18/18)	n/d	n/d
	[91]	Japan	JaGAr 01 (genotype III; *Cx. tritaeniorhynchus*, Japan 1959); JaGAr 19461 (*Cx. tritaeniorhynchu*s, Japan 1961)	n/d	n/d	n/d	n/d	Successful transmission from infected mosquitoes to uninfected lizards, and from infected lizards to mice (via mosquito)
	[181]	Japan (laboratory colony)	n/d (isolated from *Cx. annulus*, Taiwan 1972)	$10^{4.2}$ MID50 per mosquito	Parenteral infection (injection)	JEV detected at 7 dpi	n/d	n/d
	[23]	Japan, (Sapporo)	JANAr-5681 (*Culex tritaeniorhynchus*, Japan, 1981)	$10^{4.1}$ PFU/mL	viremic chick (inoculated with virus at the age of 1 d and maintained for 48–72 h)	30% at 14 dpi	n/d	n/d
	[156]	South Korea (Camp Greaves, 2003; F1 generation)	ROK-2.0028 (*Cx. tritaeniorhynchus*, South Korea, 2000)	$10^{5.2}$ PFU/mL ($10^{4.3}$ pfu/mL were not sufficient to infect successfully)	feeding on leghorn chicks (2–4 days old, inoculated with 10^2–3 PFU 2–3 days before mosquito feeding)	6% (2/32) at ≥12 dpi	0% (0/6) at ≥12 dpi	0% (0/13) [transmission to chicks]

	[168]	n/d (laboratory colony)	N28 (genotype III; pig isolate China, 2015)	10^3 MID50	Intrathoracic injection or blood meal feeding of mice blood with Hemotek system	JEV detectable at 10 dpi in whole body and midgut	n/d	JEV detectable at 10 dpi in salivary glands
	[96]	n/d (laboratory colony)	N28 (genotype III; pig isolate China, 2015)	10^3 MID50	Intrathoracic injection or blood meal feeding of mice blood with Hemotek system	JEV detectable at 10 dpi in whole body and midgut	n/d	JEV detectable at 10 dpi in salivary glands
	[167]	n/d (laboratory colony)	N28 (genotype III; pig isolate China, 2015)	10^3 MID50	Intrathoracic injection	JEV detectable at 6 dpi	n/d	n/d
Culex pipiens pipiens	[36]	USA (laboratory colonies from Yakima Valley, Washington)	Nakayama (genotype III; human, Japan, 1935)	n/d	feeding on soaked cotton moistened with mixture of 10% mouse brain virus suspension and defibrinated rabbit blood	infection proven due to virus recovery from blood-fed mosquitoes at 21 dpi	n/d	25% (1/4) at 20 dpi transmission to 21–28 day old Swiss mice (Webster strain); transmission to chicken (1/1) at 20 dpi
	[34]	France (Montpellier, 2010)	recombinant virus from strain RP-9 (genotype III; wildtype strain NT109 isolated from *Cx. tritaeniorhynchus*, Taiwan, 1985; recombinant virus from strain XZ0934 (genotype V; *Cx. tritaeniorhynchus*, China, 2009)	8 x 10^6 FFU/mL	blood meal of washed rabbit erythrocytes with 5 µM ATP, fed for 2 h via blood-soaked cotton pledges in the dark at 28 °C	26–36% at 7 dpi 70–95% at 11–13 dpi	≈25–38% at 7 dpi ≈50–80% at 11–13 dpi [measured as infected heads]	12–41% at 11–13 dpi (mean conc. for JEV RP-9: 55 FFU/saliva, for JEV XZ0934: 35 FFU/saliva)
	[98]	UK (laboratory colony)	SA14 (genotype III; Isolated from *Cx pipiens*, China, 1954)	1.8 × 10^6 pfu/mL	Blood meal feeding of defibrinated horse blood, and 1 µM ATP overnight with Hemotek system	20 ℃: 70% (39/56); 25 ℃: 90% (18/20) at 14 dpi	20 ℃: 13% (5/40); 25 ℃: 70% (14/20) at 14 dpi	20 ℃: 0% (0/40); 25 ℃: 70% (14/20) at 14 dpi
Culex pseudovishnui Culex pseudovishnui (continued)	[169]	Japan (laboratory colony, Okoyama prefecture, 1968)	JaGAr 01 (genotype III; *Cx. tritaeniorhynchus*, Japan 1959)	viremic chicken with 10^2–$10^{3.7}$ LD50/0.02 mL	feeding on viremic chicken	viremia in chicken 10^2 LD50/0.02 mL: 0% (0/19) at 10–14 dpi 10^3 LD50/0.02 mL: 11% (13/117) at 10–14	n/d	n/d

	[104]	India (laboratory colony)	P20778 (genotype III; human, India)	n/d	feeding on viremic chicks (infected with 2–3 dex virus)	n/d	60% at 8 dpi [detection of JEV antigen in mosquito heads]	44.4% [JEV antigen detection in salivary glands]; 75% (3/4) at 8 dpi [transmission to chicks]
	[105]	India (laboratory colony)	P20778 (genotype III; human, India)	4.2 log/0.2 uL MID50 for intrathoracic infection	intrathoracic injection, or oral infection via feeding of infectious blood meal	higher infection rate in intrathoracic infected compared to orally infected mosquitoes	49% (49/100) at 2-10 dpi [detection of JEV antigen in mosquito heads]	51% (57/100) at 1–10 dpi [JEV antigen detection in salivary glands]
Culex quinquefasciatus	[35,35]	Guam	Okinawa strain (human isolate, Japan, 1945)	n/d	n/d	n/d	n/d	successful transmission to infant mice
	[36]	USA	Nakayama (genotype III; human isolate, Japan, 1935)	n/d	feeding on soaked cotton moistened with mixture of 10% mouse brain virus suspension and defibrinated rabbit blood; or feeding on viremic chicken	infection proven due to virus recovery from blood-fed mosquitoes at 26 dpi	n/d	25% (4/16) at 11–25 dpi transmission to 21–28 day old Swiss mice (Webster strain) with mosquitoes infected by artificial blood meal; NO transmission to mice with mosquitoes fed on viremic chicken unsuccessful
	[108]	n/a (laboratory colony)	Okinawa strain (human isolate, Japan, 1945)	n/d	feeding on cotton soaked with virus emulsion with 10% cane sugar	n/d	n/d	100% (1/1) at 6 dpi 100% (1/) at 8 dpi [transmission to suckling mice]
	[109,170]	n/a (laboratory colony)	Okinawa strain (human isolate, Japan, 1945)	7.3 LD50	feeding on cotton soaked with virus emulsion with 10% cane sugar	n/d	n/d	100% (3/3) at 6 dpi; 72% (13/18) 13–43 dpi [transmission to mice]
	[110]	India (laboratory colony, 1954) [*Culex fatigans*]	733913 (genotype I; human, India)	4.0 dex (viremia in chicks at time point of mosquito feeding)	feeding on chicks (infected with 5.0 dex) over night	virus detected up 15 days after infection	n/d	61% (11/18) at 5–15 dpi
	[169]	Japan (laboratory colony) [*Culex pipiens fatigans*]	JaGAr 01 (genotype III; *Cx. tritaeniorhynchus*, Japan 1959)	$10^{3.9}$–10^{5} LD50/0.02 mL artificial blood meal	artificial blood meal with rabbit blood	0% (0/17) 10–14 dpi after infection with 10^{4} LD50/0.02 mL; 4% (1/24) 10–14 dpi after infection with $10^{4.3}$ LD50/0.02 mL; 38% (8/21) 10–14 dpi after infection with 10^{5} LD50/0.02 mL	n/d	n/d

	[91]	Japan [*Culex pipiens fatigans*]	JaGAr 01 (genotype III; *Cx. tritaeniorhynchus*, Japan 1959); JaGAr 19461 (*Cx. tritaeniorhynchus*, Japan 1961)	n/d	n/d	n/d	n/d	Successful transmission from infected mosquitoes to lizards, and from infected lizards to mice (via mosquito)
	[51]	India (laboratory colony) [*Cx. pipiens quinquefasciatus*]	733913 (genotype I; human, India)	n/d (chicks infected with 4.0 dex)	feeding on chicks (infected with 4.0 dex)	n/d	n/d	successful transmission to chicks
	[181]	Taiwan (laboratory colony)	n/d (isolated from *Cx. annulus*, Taiwan 1972)	$10^{4.2}$ MID50 per mosquito	Parenteral infection (injection)	JEV detected at 7 dpi	n/d	n/d
	[32]	Taiwan (Tao-Yuan)	CH1392 (genotype III; *Cx. tritaeniorhynchus*, Taiwan, 1990)	1.25×10^7 PFU/mL	fed on drop of rabbit blood (3 mL rabbit blood, 0.3 g sucrose, 1 mL virus suspension) for 1 h	n/d	n/d	40% at 14 dpi [virus detection in salivary glands]
	[171]	India	n/d	n/d	Feeding infectious blood meal	susceptibility increases when *Pseudomonas sp*. and *Acinetobacter sp*. were incorporated in the mosquito blood meal	n/d	n/d
	[38]	Australia (<u>laboratory colony</u>, Gold Coast, Queensland; and <u>field caught</u>, Mareeba, Queensland)	TS3306 (genotype II; *Och. vigilax,* Australia, 1998)	mean $10^{4.5}$ TCID50/mL	feeding for 2–4 h at 37 °C on virus diluted in heparinized rabbit blood and 1% sucrose by glass membrane feeder (pig intestine as membrane)	<u>colony</u>: 98% at 17–19 dpi in<br><br><u>field caught</u>: 56% at 14–15 dpi	<u>colony</u>: 28% at 17–19 dpi	<u>colony</u>: 50% at 17–19 dpi<br><br><u>field caught</u>: 0% at 14–15 dpi [transmission to Quackenbush suckling mice]
	[166]	<u>New Zealand</u> (field-caught, Wellington, 2009) <u>USA</u> (laboratory colony, Pennsylvania, 2008)	Nakayama (genotype III; human, Japan, 1935)	8.1 log10 pfu/mL	Feeding of virus mixed with goose, rabbit, or bovine blood, 2.5% sucrose, 0.02 µM ATP over soaked pledget	<u>New Zealand</u>: 17% (6/36) at 14 dpi<br><br><u>USA</u>: 86% (43/50) at 14 dpi	<u>New Zealand</u>: 0% (0/6) at 14 dpi<br><br><u>USA</u>: 0% (0/443) at 14 dpi	<u>New Zealand</u>: n/d<br><br><u>USA</u>: 0% (0/443) at 14 dpi

[111]	Brazil (Recife; laboratory colony)	Muar (genotype V; human, Malaysia,1952)	6 × log10 pfu/mL	Hemotek membrane feeding system (Parafilm as membrane), defibrinated horse blood supplemented with 0.02µM ATP	for 23 °C: 25% (6/24) at 7 dpi, 62% (20/32) at 14 dpi, 70% (7/10) at 21 dpi for 28 °C: 44% (4/9) at 7 dpi, 66% (8/12) at 14 dpi, 70% (7/10) at 21 dpi	for 23 °C: 21% (5/24) at 7 dpi, 34% (11/32) at 14 dpi, 70% (7/10) at 21 dpi for 28 °C: 0% (0/9) at 7 dpi, 58% (7/12) at 14 dpi, 70% (7/10) at 21 dpi	for 23 °C: 17% (4/24) at 7 dpi, 3% (1/32) at 14 dpi, 50% (5/10) at 21 dpi for 28 °C: 0% (0/9) at 7 dpi, 17% (2/12) at 14 dpi, 70% (7/10) at 21 dpi
[112]	USA (Valdosta, Georgia)	Tiara (genotype III; human, Japan, 1948)	$10^{4.8}$ TCID50/mL (in engorged mosquitoes immediately after infection/ feeding)	Feedings with Hemotek feeders and blood-soak cotton pledges for 1 h	100% at 7 dpi (mean conc.: $10^{4.2}$ TCID50/mL) 84.6% at 14 dpi (mean conc.: $10^{4.5}$ TCID50/mL)	0% at 7 dpi 50% at 14 dpi	9.1% at 14 dpi
[172]	n/d	057434 (genotype III; human, India, 2005)	100 pfu per mosquito	Intrathoracic injection or feeding of infectious blood meal	JEV detectable at 7 dpi and 10 dpi	n/d	n/d
[113]	USA (Valdosta, Georgia; F6 generation)	KE-93–83 (genotype Ia; mosquitoes, Thailand, 1983); JE-91 (genotype Ib; mosquitoes, Korea, 1991); Taira (genotype III; human, 1948, Japan)	$10^{7.99}$–$10^{8.36}$ TCID50/mL in blood meal ($10^{4.36}$–$10^{4.81}$ TCID50/mL per engorged mosquitoes)	Feedings with Hemotek feeders and blood-soak cotton pledges with defibrinated sheep blood for 1 h	44–95% at 7 dpi (total: 76/115) 35–67% at 14 dpi (total: 88/165)	8–30% at 7 dpi (total: 8/47) 17–32% at 14 dpi (total: 15/54)	5–8% at 14 dpi (total: 6/88)

	[114]	USA (laboratory colony, Florida, 1988)	KE-093–83 (genotype Ia; mosquitoes, Thailand, 1983); MAR864 (genotype I; *Cx. tritaeniorhynchus*, Cambodia, 1967); JE-91 (genotype Ib; *Cx. tritaeniorhynchus*, Korea, 1991); CH392 (genotype III; *Cx. tritaeniorhynchus*, Taiwan, 1987); JKT27–087 (genotype III; mosquitoes, Indonesia, 1987); Sagiyama (genotype III; *Cx. tritaeniorhynchus*, 1957, Japan)	5.1–5.9 log10 pfu/mL	Feeding of defibrinated cattle blood mixed with freshly harvested viruses at a 1:1 via Hemotek membrane feeding system covered with pork casing	KE-093–83: 93% (56/60) at 7 dpi, 78% (47/60) at 14 dpi; MAR864: 93% (56/60) at 7 dpi, 82% (49/60) at 14 dpi; JE-91: 78% (47/60) at 7 dpi, 97% (58/60) at 14 dpi; CH392: 40% (24/60) at 7 dpi, 22% (13/60) at 14 dpi; JKT27–087: 58% (35/60) at 7 dpi, 70% (42/60) at 14 dpi; Sagiyama: 53% (32/60) at 7 dpi, 85% (51/60) at 14 dpi	KE-093–83: 30% (18/60) at 7 dpi, 55% (33/60) at 14 dpi; MAR864: 35% (21/60) at 7 dpi, 18% (11/60) at 14 dpi; JE-91: 43% (26/60) at 7 dpi, 72% (43/60) at 14 dpi; CH392: 25% (15/60) at 7 dpi, 17% (10/60) at 14 dpi; JKT27–087: 8% (5/60) at 7 dpi, 12% (7/60) at 14 dpi; Sagiyama: 32% (19/60) at 7 dpi, 22% (13/60) at 14 dpi	KE-093–83: 2% (1/60) at 7 dpi, 7% (4/60) at 14 dpi; MAR864: 13% (8/60) at 7 dpi, 8% (5/60) at 14 dpi; JE-91: 5% (3/60) at 7 dpi, 18% (11/60) at 14 dpi; CH392: 0% (0/60) at 7 dpi, 5% (3/60) at 14 dpi; JKT27–087: 0% (0/60) at 7 dpi, 3% (2/60) at 14 dpi; Sagiyama: 0% (0/60) at 7 dpi, 7% (4/60) at 14 dpi
Culex sitiens	[38]	Australia (laboratory colony, Coomera Island, Queensland)	TS3306 (genotype II; *Och. vigilax,* Australia, 1998)	mean $10^{4.5}$ TCID50/mL	feeding for 2–4 h at 37 °C on virus diluted in heparinized rabbit blood and 1% sucrose by glass membrane feeder (pig intestine as membrane)	83% (15/18) at 5 dpi, 83% (15/18) at 7 dpi, 89% (16/18) at 10 dpi, 92% (33/36) at 14 dpi	6% (1/18) at 5 dpi, 28% (5/18) at 7 dpi, 33% (6/18) at 10 dpi, 11% (4/36) at 14 dpi	13% (2/15) at 7 dpi, 7% (1/15) at 10 dpi, 67% (10/15) at 14 dpi [transmission to Quackenbush suckling mice]

Culex tarsalis	[36]	USA	Nakayama (genotype III; human isolate, Japan, 1935)	n/d	feeding of mixture of 10% mouse brain virus suspension and defibrinated rabbit blood on soaked cotton	infection proven due to virus recovery from blood-fed mosquitoes at 15–25 dpi	n/d	6% (2/35) at 6–10 dpi [transmission to 21–28 day old Swiss mice (Webster strain)]
Culex tritaeniorhynchus	[148]	Japan (field caught)	n/d	n/d	mosquito naturally infected and directly used for transmission experiments to mice	n/d	n/d	successful transmission to mice
	[90]	n/d	Nakayama (genotype III; human isolate, Japan, 1935)	n/d	feeding of mouse-brain-virus solution in 20% saline and defibrinated rabbit blood over cotton pads	n/d	n/d	100% (3/3) at 6–7 dpi [transmission to mice]
	[149]	Singapore [*Culex tritaeniorhynchus siamensis*]	local isolate (not further determined); Nakayama (genotype III; human, Japan, 1935)	100 LD50 per mosquito	Infected mouse-brain suspensions in defibrinated rabbit blood containing a little cane-sugar solution	n/d	n/d	transmission to chicks:
	[150]	Japan (Tokyo, 1955; F12-F17 generations)	M5/596 (*Cx. tritaeniorhynchus*, Japan, 1955)	$10^{2.3}$–$10^{3.3}$ LD50/0.04 mL	feeding on infected pigs; or white Leghorn chicks (infected with 160,000 mice LD50)	feeding in viremic pigs: 55% (16/29) at 10–14 dpi, 37% (11/30) at 15–19 dpi; feeding on viremic chicks: 56% (62/111) at 10–14 dpi, 66% (47/71) at 15–19 dpi	n/d	pig-pig transmission: 33% (2/6) at 15–19 dpi, 33% (4/12) at 20–24 dpi; chick-chick transmission: 28% (8/29) at 15–19 dpi, 21% (4/19) at 20–24 dpi; additional successful transmission to Black-crowned Night Herons, Plumed Egrets, and Great Egret
	[161]	Japan (laboratory strain)	T-143 (*Cx. tritaeniorhynchus*, Taipei, Taiwan, 1960)	viremia in pigs $10^{0.1}$–$10^{2.5}$ LD/50	feeding on viremic pigs	6% (17/293) when viremia 10^{01} –10^{1} LD50; 14% (34/237) when viremia $10^{1.1}$–10^{2} LD50; 45% (48/107) when viremia $10^{2.1}$–10^{3} LD50	n/d	n/d

[151]	Japan (laboratory colony)	FM380 (*Cx. tritaeniorhynchus*, Japan, 1950)	n/d (viremia in chicks around 10^4 LD50)	3–7 days after emergence feeding on viremic chicks for transmission to horses; feeding on infected horse for further transmission to chicks	70% (47/67) after feeding on viremic chicks	n/d	67% (2/3) [transmission to horses] 2% (1/62) [transmission to chicks; mosquitoes fed on infected horses]
[184]	Japan (laboratory colony) [*Culex tritaeniorhynchus summorosus*]	n/d	n/d	Feeding on viremic chicks	JEV detected from 5 dpi on in midgut cells	JEV detected from 9 dpi on in fat body	JEV detected from 12 dpi on in salivary glands
[152]	India	9215 (*Cx. tritaeniorhymchus*, India 1964)	n/d	feeding on pig for 1 h	65% at 14 dpi (total:148/228)	n/d	14% (2/14) [transmission to suckling mice]; 64% (25/39) [virus detection in saliva]; 67% (2/3) [transmission to pigs]
[185]	n/d (laboratory colony)	Attenuated m strains of Mukai (human, Japan),	*blood meal*: 1.5–3.9 TCID50 *chicken:* 4.9–6.2 SMICLD50	Feeding blood meal with fresh heparinized rabbit blood through nylon gauze, and feeding on infected chickens (white Leghorn)	*blood meal*: NO infection with attenuated JEV; *Chicken:* 100% (18/18) with Mukai strain; 0% (22/22) with highly attenuated m strain	n/d	n/d
[60]	Thailand (Chiengmai valley)	BKM-984–70 (*Cx. fuscocephala*, Thailand, 1970)	average 8 pfu per mosquito	feeding on viremic white Leghorn chicken (infected with 1700 pfu 55 h before mosquito feeding)	18–20% at 10–27 dpi	n/d	10–40% at 11–27 dpi [transmission to chicks]
[61]	Taiwan (JEVRU colony)	TaiAn 171 (pig, Taiwan, 1971)	$10^{0.89}$–$10^{1.91}$ LD50 in pig serum on day of mosquito feeding	feeding on viremic pigs (infected with $10^{5.5}$ mouse LD50)	n/d	n/d	0% at 12 dpi 10% at 17 dpi 5% at 18 dpi [transmission to white leghorn chicks]
[153]	Japan (laboratory colony, Tokyo)	JaGAr 01 (genotype III; *Cx. tritaeniorhynchus*, Japan 1959)	$10^{1.7}$ LD50 per blood meal	artificial membrane feeding with heparinized rabbit blood (Nylon gauze as membrane)	when fed with >10^4 LD50/0.002 mL then infection rates approaching 100% could be expected	n/d	virus detected in salivary glands 6–24 dpi, and in saliva from 10–20 dpi

[110]	India (laboratory colony, 1967)	733913 (genotype I; human, India)	4.0 dex (viremia in chicks at time point of mosquito feeding)	feeding on chicks (infected with 5.0 dex) over night	n/d	n/d	successful transmission to chicks
[154]	India (laboratory colony, Virus Research Centre)	n/d (*Cx. whitmorei*, India)	1.2–2.5 dex LD50	feeding on viremic Ardeid birds (*Bubulcus ibis*, *Ardeola grayii*) or white Leghorn chicks (infected with 2.7–3.3 dex LD50)	n/d	n/d	100% (2/2) at 8–11 dpi [transmission to to *Ardeola grayii*] 67% (2/3) at 8–11 dpi [transmission to white Leghorn chicks]
[49]	India (Pune, laboratory colony)	733913 (genotype I; human, India)	n/d (ducklings infected with 4.9–5.2 dex)	feeding in viremic ducklings (viremia 1–4.5 dex)	41% (11/27) at 9–12 dpi	n/d	100% (7/7) [transmission to ducklings]
[169]	Japan (laboratory colony) [*Culex tritaeniorhynchus summorosus*]	JaGAr 01 (genotype III; *Cx. tritaeniorhynchus*, Japan 1959)	viremic chicken with 10^3 icLD50/0.02 mL	feeding on viremic chicken	90% (36/40) 10–14 dpi	n/d	n/d
[183]	Japan (laboratory colony)	JaGAr 01 (genotype III; *Cx. tritaeniorhynchus*, Japan 1959)	n/d	intrathoracic injection or blood meal feeding with defibrinated rabbit blood through nylon-gauze membrane	n/d	n/d	JEV detected in salivary glands 26 dpi (intrathoracic injection), or 14 dpi (membrane feeding)
[174]	Japan (laboratory colonies from Kyoto, 1966; Osaka, 1976; Okinawa, 1970; Miyagi, 1970; Nagasaki, 1965; Amami, 1972); and Taiwan (laboratory colony, Taipei, 1971)	JaGAr 01 (genotype III; *Cx. tritaeniorhynchus*, Japan 1959)	$10^{1.7}$–$10^{2.6}$ LD50/0.03 mL	artificial membrane feeding with defibrinated rabbit blood	Kyoto: 30–60%; Osaka: 50–60%; Okinawa: 60%; Miyagi: 50%; Nagasaki: 40%; Amami: 95%; Taiwan: 15%	n/d	n/d

[155]	Taiwan (colony)	JE 2–8 (vaccine strain originated from strain SA-14)<br>SA-14 (genotype III; *Cx. tritaeniorhynchus*, China, 1960)	$10^{2.7}$–10^{3} TCID50/mL for oral infection;<br>10^{5} TCID50/mL for intrathoracic infection	oral infection with human red blood cells, calf serum and sucrose, feeding with cotton pledges;<br>or<br>intrathoracic injection	<u>oral infection</u><br>at 12–15 dpi<br>SA-14:<br>100% (19/19)<br>(mean conc.: $10^{5.7}$ TCID50/mosquito)<br>JE 2–8:<br>11% (4/36)<br>(mean conc.: 10^{1} TCID50/mosquito)<br><br>intrathoracic infection at 10 dpi<br>SA-14:<br>100% (45/45)<br>JE 2–8:<br>100% (35/35)	n/d	<u>oral infection</u><br>at 12–15 dpi<br>SA-14:<br>76% (total: 26/34)<br>JE 2–8:<br>0% (total: 0/37) at<br><br><u>intrathoracic infection</u><br>at 12–15 dpi<br>SA-14:<br>100% (total: 46/46)<br>JE 2–8:<br>3% (total: 1/36)<br>[transmission to mice]
[51]	India (laboratory colony)	733913 (genotype I; human, India)	n/d (chicks infected with 4.0 dex)	feeding on chicks	n/d	n/d	successful transmission to chicks
[181]	Japan (laboratory colony)	n/d (isolated from *Cx. annulus*, Taiwan 1972)	$10^{4.2}$ MID50 per mosquito	Parenteral infection (injection)	JEV detected at 7 dpi	n/d	n/d
[186]	China (field caught, F0, Beijing)	A2	n/d	Feeding on infected Swiss mice	83% (10/12) at 15 dpi;<br>*Co-infection with M14 virus:*<br>8% (1/12) at 10–15 dpi	n/d	n/d
[187]	Thailand (field caught, F1, Bang Pa-In))	KE094 (genotype I; human, Thailand, 1983);<br>34–35 CT (genotype III; isolated from *Cx. tritaeniorhynchus*, Thailand, 1982)	1 pfu per mosquito	Intrathoracic injection	JEV detected in fat body from 2 dpi on	JEV detected in heads from 2 dpi on	JEV detected in salivary glands from 4 dpi on (when mosquitoes held at 32 ℃)
[23]	Japan, (Sapporo)	JANAr-5681 (*Cx. tritaeniorhynchus*, Japan, 1981)	n/d	virus-blood mixture soaked cotton	100% at 14 dpi	n/d	100% at 14 dpi [transmission to mice]
[188]	Thailand	Nakayama (genotype III; human, Japan, 1935)	$10^{9.4}$ MID50	Intrathoracic injection	JEV antigen detection 15 dpi	n/d	n/d

[31]	Taiwan (laboratory colony, Petou strain, Taipei)	MQ1–2 (*Ae. albopictus*, Taipei, Taiwan, 1985)	6.5 log10 (LD50/0.03 mL)	n/d	successful infection, median virus conc. 1.02 log10 MID50	n/d	n/d
[92]	Taiwan (Taipei; F30–31 generation)	Sanhsia MQ1–2 (*Ae. albopictus*, Taiwan, 1995)	$10^{4.5}$ pfu/mL (ingested $10^{5.48}$ pfu/mL per mosquito); and 10^3 to 10^8 pfu/mL	feeding on hanging drop of defibrinated rabbit blood with 10% of sucrose for 3 h	n/d	n/d	100% (total: 20/20) [transmission to mice]
[105]	India (laboratory colony)	P20778 (genotype III; human isolate, India)	n/d	intrathoracic injection, or oral infection via feeding of infectious blood meal	higher infection rate in intrathoracic ally infected compared to orally infected mosquitoes	51% (51/100) at 2–10 dpi [detection of JEV antigen in mosquito heads]	57% (57/100) at 1–10 dpi [JEV antigen detection in salivary glands]
[162]	Taiwan (laboratory colony)	SA14 (genotype III; Isolated from *Cx. pipiens*, China, 1954)	6.0 $\log_{10}$ pfu/mL (5.5 $\log_{10}$ pfu per mosquito) for intrathoracic injection	Intrathoracic injection and feeding blood meal with washed calf red blood cells with membrane feeder for 15–30 min	*Intrathoracic injection:* at 14 dpi 6.7 $\log_{10}$ per mosquito; *Orally infected:* at 15 dpi 7.8 $\log_{10}$ pfu per mosquito	JEV detected in heads	n/d
[156]	South Korea (Camp Greaves, 2003; F1 generation)	ROK-2.0028 (*Cx. tritaeniorhynchus*, South Korea, 2000)	10^4.3 - 10^5.2 pfu/mL	feeding on leghorn chicks (2–4 days old, inoculated with 10^2–3 pfu 2–3 days before mosquito feeding)	100% (24/24) at ≥12 dpi	88% (21/24) at ≥12 dpi	67% (4/6) [transmission to chicks]; 33% (5/15) [virus detected in saliva]
[172]	n/d	057434 (genotype III; human, India, 2005)	100 pfu per mosquito	Intrathoracic injection or feeding of infectious blood meal	JEV detectable at 7 dpi and 10 dpi	n/d	n/d
[157]	Japan (laboratory colony, Shimane prefecture, 2018)	17CxIT-I4-D31 (genotype I, *Cx. tritaeniorhynchus*, Japan, 2017); JaGAr01 (genotype III, *Cx. tritaeniorhynchus*, Japan, 1959); Muar (genotype V, human, Malaysia, 1952)	17CxIT-I4-D31: 8.9 log10 ffu/mL; JaGAr01: 8.6 log10 ffu/mL; Muar: 7.1 log10 ffu/mL	Feeding defibrinated rabbit blood 1:1 mixed with virus, supplemented with 3 mM ATP via Hemotek feeder (pig intestine as membrane)	17CxIT-I4-D31: 77% (27/35) at 7 dpi, 92% (37/40) at 14 dpi; JaGAr01: 100% (49/49) at 7 dpi, 98% (48/49) at 14 dpi; Muar: 100% (51/51) at 7 dpi, 98% (49/50) at 14 dpi	17CxIT-I4-D31: 74% (26/35) at 7 dpi, 88% (35/40) at 14 dpi; JaGAr01: 94% (46/49) at 7 dpi, 98% (48/49) at 14 dpi; Muar: 96% (49/51) at 7 dpi, 96% (48/50) at 14 dpi	17CxIT-I4-D31: 85% (23/27) at 7 dpi, 92% (34/37) at 14 dpi; JaGAr01: 78% (38/49) at 7 dpi, 92% (44/48) at 14 dpi; Muar: 88% (45/51) at 7 dpi, 92% (45/49) at 14 dpi

Culex vishnui	[105]	India (laboratory colony)	P20778 (genotype III; human, India)	n/d	intrathoracic injection, or oral infection via feeding of infectious blood meal	higher infection rate in intrathoracic infected compared to orally infected mosquitoes	34% (34/100) at 2–10 dpi [detection of JEV antigen in mosquito heads]	48% (48/100) at 1–10 dpi [JEV antigen detection in salivary glands]
Culiseta annulata	[95]	UK (field caught, F1, Cheshire)	CNS138–11 (genotype II, human, Malaysia, 1999)	1 × 10^6 pfu/mL	Feeding of heparinised human blood (heated to 39 ℃) with Hemotek feeding system for up to 3 h	21 ℃: 43% (13/30) at 14 dpi; 57% (20/35) at 21 dpi; 10% (3/30) at 28 dpi 24 ℃: 20% (6/30) at 14 dpi; 17% (4/24) at 21 dpi; 0% (0/5) at 28 dpi	n/d	21 ℃: 30% (9/30) at 14 dpi; 20% (15/35) at 21 dpi; 3% (1/30) at 28 dpi 24 ℃: 0% (0/30) at 14 dpi; 0% (0/24) at 21 dpi; 0% (0/5) at 28 dpi
Culiseta incidens	[36]	USA	Nakayama (genotype III; human, Japan, 1935)	n/d	feeding of mixture of 10% mouse brain virus suspension and defibrinated rabbit blood on soaked cotton	infection proven due to virus recovery from blood-fed mosquitoes at 17 and 20 dpi	n/d	no transmission to 21–28 day old Swiss mice (Webster strain)
Culiseta inornata	[36]	USA	Nakayama (genotype III; human, Japan, 1935)	n/d	feeding of mixture of 10% mouse brain virus suspension and defibrinated rabbit blood on soaked cotton	infection proven due to virus recovery from blood-fed mosquitoes at 20 dpi	n/d	19% (3/16) [transmission to 21–28 day old Swiss mice (Webster strain)]
Heizmannia taiwanensis	[181]	Taiwan (laboratory colony, Hsinchu)	n/d (isolated from *Cx. annulus*, Taiwan 1972)	$10^{4.2}$ MID50 per mosquito	Parenteral infection (injection)	JEV detected at 7 dpi	n/d	n/d
Opifex fuscus	[166]	New Zealand (laboratory colony, Wellington, 2007)	Nakayama (genotype III; human, Japan, 1935)	8.1 log10 pfu/mL	Feeding of virus mixed with goose, rabbit, or bovine blood, 2.5% sucrose, 0.02 µM ATP over soaked pledget	74% (37/50) at 14 dpi	70% (26/37) at 14 dpi	0% (0/37) at 14 dpi
Mansonia septempunctata	[38]	Australia (field caught, Cairns, Queensland)	TS3306 (genotype II; *Och. vigilax,* Australia, 1998)	mean $10^{4.5}$ TCID50/mL	feeding for 2–4 h at 37 °C on virus diluted in heparinized rabbit blood and 1% sucrose by glass membrane feeder (pig intestine as membrane)	67% (16/24) at 14–15 dpi	54% (13/24) at 14–15 dpi [virus detection in legs, and head + salivary glands]	n/d
Mansonia uniformis	[38]	Australia (field caught, Cairns, Queensland)	TS3306 (genotype II; *Och. vigilax,* Australia, 1998)	mean $10^{4.5}$ TCID50/mL	feeding for 2–4 h at 37°C on virus diluted in heparinized rabbit blood and 1% sucrose by glass membrane feeder (pig intestine as membrane)	100% (1/1) at 14–15 dpi	100% (1/1) at 14–15 dpi [virus detection in legs, and head + salivary glands]	n/d

Topomyia yanbarensis	[181]	Taiwan (laboratory colony, Taipei)	n/d (isolated from *Cx. annulus*, Taiwan 1972)	$10^{4.2}$ MID50 per mosquito	Parenteral infection (injection)	JEV detected at 7 dpi	n/d	n/d
Tripteroides bambusa	[181]	Taiwan (laboratory colony, Taitung)	n/d (isolated from *Cx. annulus*, Taiwan 1972)	$10^{4.2}$ MID50 per mosquito	Parenteral infection (injection)	JEV detected at 7 dpi	n/d	n/d
Verrallina funerea	[38]	Australia (field caught, Cairns, Queensland)	TS3306 (genotype II; *Och. vigilax,* Australia, 1998)	mean $10^{4.5}$ TCID50/mL	feeding for 2–4 h at 37 °C on virus diluted in heparinized rabbit blood and 1% sucrose by glass membrane feeder (pig intestine as membrane)	11% (4/36) at 14–15 dpi	n/d	7% (1/15) at 14–15 dpi [transmission to Quackenbush suckling mice]

* Species name (without subspecies), outdated species named in []; & Genotype and isolation details in brackets; # Detection of virus in saliva if not mentioned otherwise; TCID50 50% tissue infectious dose; WMLC50 50% weaning mice intracranial lethal dose; LC50 50% lethal dose; pfu plaque forming unit; ffu foci forming unit; dpi days post infection (exposure); n/d not determined.
